# Supplementary material for: A high-resolution mRNA expression time course of embryonic development in zebrafish
Source: eLife. 2017 Nov 16;6:e30860. doi: 10.7554/eLife.30860 (PMC5690287; doi:10.7554/eLife.30860)
Supplement: Supplementary file 6. [file elife-30860-supp6.zip › biolayout-clusters-files/Cluster185-genes.html]

Cluster185


# Cluster185: Genes

| Ensembl ID | Gene Name | Chr | Start | End | Biotype |
| --- | --- | --- | --- | --- | --- |
| ENSDARG00000029370 | ankrd6b | 20 | 1182326 | 1218777 | protein\_coding |
| ENSDARG00000053857 | ccdc187 | 23 | 30086709 | 30119202 | protein\_coding |
| ENSDARG00000102474 | dusp16 | 4 | 149700 | 334996 | protein\_coding |
| ENSDARG00000075964 | plekhg4 | 7 | 34417055 | 34505422 | protein\_coding |
| ENSDARG00000078748 | si:ch211-137a8.4 | 15 | 29153079 | 29158931 | protein\_coding |
| ENSDARG00000045706 | tspan9b | 4 | 9696049 | 9721652 | protein\_coding |
| ENSDARG00000034670 | ube2e2 | 16 | 49797003 | 49866653 | protein\_coding |
| ENSDARG00000033327 | unc5b | 13 | 28997628 | 29097341 | protein\_coding |
